# Autism care: reimagining the spectrum

**DOI:** 10.3389/frcha.2026.1825292

**Published:** 2026-08-20

**Authors:** Steven Merahn

**Affiliations:** Perimeter Healthcare, Alpharetta, GA, United States

**Keywords:** autism spectrum disorder, neuro-affirming practice, spectrum of care, preventive intervention, applied behavior analysis, integrated care

## Abstract

Autism care policy is at a critical inflection point. Applied behavior analysis (ABA), long established as the “gold standard” through state insurance mandates in the US, has functioned as the default reimbursable intervention for autistic children. However, advances in genomics, neuroscience, developmental psychology, and scholarship on autistic lived experience have expanded understanding of autism as a heterogeneous neurotype characterized by meaningful differences in neural organization rather than a unitary disorder. Contemporary models emphasize neurodiversity, strengths-based perspectives, and the interaction between developmental processes and environmental contexts in shaping functional outcomes. Many autistic children also meet criteria for complex care needs, requiring coordinated, interdisciplinary services across health, educational, and community systems. This manuscript proposes reframing the “autism spectrum” from a hierarchy of symptom severity to a prevention-oriented “spectrum of care.” Adapting a public health taxonomy, interventions are organized into universal, selective, and indicated levels, targeting the prevention of avoidable disability, distress, and participation barriers. This model aligns autism services with whole-child, neurodiversity-affirming, and developmentally informed care, emphasizing relational health, autonomy, and life-course participation.

## Introduction

Autism care policy is at a critical inflection point. For decades, applied behavior analysis (ABA) has been promoted as the “gold standard” intervention framework (1), a status that emerged in the United States through the efforts of parents and advocates who, dissatisfied with outcomes associated with earlier approaches to autism care, organized state-by-state legislative campaigns to secure coverage. The effort ensured all 50 states enacted some form of ABA insurance mandate (2), effectively institutionalizing ABA as the default reimbursable intervention for autistic children in the United States.

However, the “gold standard” characterization does not correspond to a single citable reference in the peer-reviewed medical literature, nor does it reflect the conclusions of rigorous independent evidence reviews. A 2018 Cochrane review of Early Intensive Behavioral Intervention rated the available evidence as “low to very low” (3), and a 2023 meta-analysis found that when progressive methodological bias restrictions were sequentially applied to over 250 studies, effects attributable to traditional ABA did not survive (4). Further diluting ABA's role in the armamentarium is state-level regulatory oversight revealing inconsistent quality of care delivery and standards of training and credentialing, and a lack of consensus on rigorous, clinically meaningful outcome measures that would allow payers or families to evaluate real-world effectiveness. The commercial structure of ABA services—built around reimbursement models that incentivize high service hours—has aligned financial incentives with volume rather than outcomes, yet accumulating evidence raises serious questions about whether greater treatment intensity is associated with meaningfully better results in communication, adaptive behavior, or social functioning (5), and there is growing concern over a potential association between ABA, masking, and long-term mental health (6). Taken together, these factors explain not only how ABA achieved its statutory status, but why that status is no longer clinically or ethically defensible as a presumptive default.

However, in the last decade, driven by rising prevalence and policy attention, the evidence base informing autism care has expanded substantially, creating new opportunities to expand the perspective on autism care. Advances in genomics (7), neuroscience (8), developmental psychology (9), and growing neurodiversity scholarship on the positive effects of autistic identity and neuro-affirming practices on life-course outcomes (10) have yielded a more sophisticated understanding of autism, and the services and supports autistic children and young adults require. From this growing body of knowledge, a set of principles has emerged that will reshape what constitutes a “gold standard” for autism care.

## Understanding autistic experience

First, autism is increasingly understood not as a single condition but as a neurotype (11), characterized by diverse differences in neural networks that influence how individuals perceive, prioritize, and regulate internal and external information across sensory, cognitive, emotional, and social domains. Differences involving the default mode network (12), salience network (13), interoception (14), systematizing tendencies (15), and the frontoparietal control network (16) are now understood as developmentally and neuropsychologically meaningful rather than inherently pathological (17). These differences imply an alternative organization of self-experience with implications for how autistic individuals navigate engagement, participation, meaning, and identity. This perspective deepens understanding of the heterogeneity of the autistic phenotype—commonly referred to as “the spectrum.”

Two examples illustrate how these findings could directly inform care design. Research on interoceptive differences in autism—the ability to perceive and interpret internal bodily signals—helps explain both emotional dysregulation and atypical pain or hunger responses, and points toward universal supports such as structured body-awareness routines and co-regulatory adult practices rather than behavioral correction (18). Similarly, the transition to middle school—where escalating social complexity, sensory load, and performance demands are predictable and universal (19)—offers a concrete context for selective intervention: anticipatory guidance, environmental preparation, and stress-monitoring, which, based on what we know about differences in salience network function (20), can proactively attenuate risk before distress becomes crisis (21).

Second, this framework has informed a more nuanced understanding of neurodiversity. Many of the cognitive and perceptual strengths associated with autism—such as attention to detail, pattern recognition, deep focus, honesty, persistence, and creativity—arise from the same neural architecture that underlies diagnosis-related symptoms and behavior (22). Recognizing this interdependence challenges deficit-only models and reframes intervention goals.

Third, there has been a shift in how autism-related disability is conceptualized. Contemporary models emphasize the interaction between developmental processes and environmental contexts as critical determinants of functional outcomes (23). This perspective does not minimize the presence of significant challenges but reframes intervention priorities toward relational health, communication, well-being, autonomy, and sustainable participation in family and community life. These principles now inform neurodiversity-affirming practices across healthcare (24), education (25), employment (26), and community settings.

Finally, many autistic children meet established criteria for children with complex care needs (27): chronic functional and developmental challenges and co-occurring physical and mental health conditions, involvement of multiple service systems, and high levels of support required by both children and families. Pediatric evidence consistently demonstrates that effective care for complex, multifactorial conditions integrates multiple therapeutic modalities (28), actively involves caregivers, and coordinates developmental, behavioral, mental health, speech, occupational, and educational supports.

Taken together, these principles indicate that the “gold standard” of autism care cannot be grounded in any single discipline or intervention model, but rather in the capacity of systems and clinicians to care for autistic children as whole people. To avoid what author Rebecca Solnit has termed “the tyranny of the quantifiable” (29), individualized care planning must extend beyond discrete skill acquisition or behavior management to encompass personhood, meaning, purpose, and contribution within families and communities.

With this foundation, the concept of the “autism spectrum” can be reframed—not as a hierarchy of symptoms or severity—but as a spectrum of care.

## A new spectrum of care

Although the spectrum construct was originally proposed to reflect autism's phenotypic diversity, it has notable limitations as a framework for guiding services and supports (30) across developmental trajectories, functional needs, and environmental contexts that influence life-course outcomes.

To address this gap, an operational classification scheme adapted from public health prevention (31) can be applied (see Table 1). This approach organizes interventions into universal, selective, and indicated levels based on population needs and complexity. The reframing proposed here is not without precedent: depression is best managed through a tiered model that maps directly onto this framework and then escalates further through a stepped care model as patient complexity evolves (32). A similar tiered escalation structure exists in the Kaiser Permanente Chronic Care Management pyramid, which stratifies patients with conditions like diabetes, asthma, and heart failure into three levels (33).

**Table 1:** Examples of services and supports at each level of the autism care spectrum as per the revised Gordon operational classification (31).

		INDICATED: Identified Risks/Conditions
	SELECTIVE: Predictable Contextual Risk	
UNIVERSAL: Foundational Supports		
Neurodiversity-affirming environments Inclusive communications support Predictable, sensory-sensitive environments Relational regulation and stress tolerance Health-promoting daily rhythms (sleep, nutrition, movement) Belonging, safety, autonomy Developmental scaffolding	Transition- and setting-focused environmental and developmental supports Targeted communication supports Stress anticipation and demand-pacing strategies Identity, mental health, and social protection Targeted sleep, feeding, and physiologic supports Context-specific safety and family system supports	Individualized environmental accommodations Intensive functional and communication supports Targeted regulation and mental health treatment Condition-specific medical/interdisciplinary care Safety-focused injury and harm prevention Intensive family system and care coordination supports

Supports are additive, and escalate based on evolving child-context complexity and risk, not autism severity.

When reframed for autism from a whole-child perspective, this taxonomy shifts the target of intervention away from autism itself and toward the prevention of avoidable disability, distress and trauma (34), co-occurring health and mental health conditions, and participation barriers that emerge from interactions among individual characteristics, communication and sensory differences, and social environments.

Universal interventions are appropriate for all autistic children, regardless of age, diagnostic presentation or perceived severity. These can include emphasis on relational health and strategies that promote regulation and acceptance; caregiver mediated inclusive communication practices; establishing neurodiversity-affirming environments with sensory accessibility and predictable routines; accessible communication supports such as visual aids and processing time; and experiences that support connection and belonging. Universal approaches parallel well-established preventive practices (e.g., seatbelt use, flu vaccines, fluoride in drinking water) and aim to reduce baseline risk for later impairment while enhancing participation in daily activities.

Selective interventions target subgroups of autistic children distinguished by non-clinical demographic, sociographic or life-stage factors such as age, gender, geography, culture, or contextual risk factors rather than diagnostic severity alone. Examples include universal autism screening in early childhood, children experiencing transitions such as school entry or puberty, or entering young adulthood; autistic girls and gender-diverse youth who may mask needs at substantial psychological cost; children in high-demand or under-resourced environments, or who are otherwise medically complex. These interventions emphasize anticipatory guidance, transition planning, stress monitoring, and contextual coaching for caregivers and educators, consistent with pediatric models recognizing the interdependence of development and environment. Selective approaches parallel public health prevention strategies such as lung cancer screening in smokers, colonoscopy in adults over age 45, and HPV vaccination in adolescents, and aim to reduce baseline risk for downstream consequences while enhancing participation in daily activities.

Indicated interventions focus on targeting specific risks or conditions, including significant barriers to developmental progress or communication, acquisition of essential daily living skills, safety risks, self-injurious behavior, aggression, feeding disorders such as avoidant/restrictive food intake disorder, sleep disorders, mental health diagnoses, and co-occurring intellectual disability. These conditions are often emerging from the interaction of developmental vulnerability, unmet support needs, environmental demands over time, or are co-occurring, rather than as intrinsic features of autism itself. They require individualized, interdisciplinary care that minimizes harm and prioritizes autonomy, communication access, and trauma-informed practice. A growing portfolio of interventions are applicable within this framework, with ABA appropriate for defined use cases in the context of the broader approach to care.

This prevention-oriented spectrum of care offers several advantages. It avoids pathologizing neurodivergent traits while clearly delineating preventive, habilitative, rehabilitative, and therapeutic services as medically necessary. It reflects the dynamic, developmental nature of risk and need, and it supports equitable resource allocation by justifying investments in universal and selective supports that may reduce reliance on crisis-driven or intensive interventions later.

## Reimagining autism care

Reimagining the autism spectrum as a model of care is a beginning, not an endpoint. Translating this framework into practice will require a disciplined, coordinated, multistakeholder effort—one that must include the voices and lived experience of autistic people themselves—to reach consensus on how specific interventions, services, and supports are allocated across the three levels of care.

Workforce preparation will be foundational. Pediatricians, educators and school staff, and community providers will need grounding in neurodiversity-affirming principles and the developmental science that underlies them. Eligibility criteria, competency standards, and fidelity monitoring will be needed across selective and indicated services to prevent the same drift toward volume-driven, poorly regulated care that has undermined the current system.

Accountability must be designed in from the start. Meaningful outcome metrics should encompass relational health, psychological and physical well-being, communication capacity, cognitive engagement and learning, and family functioning—not only discrete skill acquisition—and must be stratified by race, geography, and family resources to surface potential disparities in who is benefiting. Payers will need updated coverage frameworks that recognize universal and selective supports as medically necessary, but because this model distributes care more broadly across health, educational, workplace, and community settings, additional funding considerations will be required. Importantly, the structure of this framework also lends itself to quality measurement: access to universal and selective interventions can be tracked at the population level, and indicated services can be benchmarked against clearly defined eligibility criteria.

The proposed Spectrum of Care framework provides a coherent, evidence-based foundation for neurodiversity-informed whole-child care that accounts for both autistic identity and the full range of support needs across the population. By emphasizing upstream developmental prevention, reducing avoidable harm, and increasing participation across diverse disciplines and settings, this framework aligns clinical practice with emerging standards for complex care and establishes a structure for population-based quality measures. Most importantly, it strengthens our capacity to support autistic children throughout their life-course and have more opportunities to succeed in the world on their own terms.

## Data Availability

Data sharing is not applicable to this article as no datasets were generated or analyzed during the current study. Further inquiries can be directed to the corresponding author.

## References

[1] Murillo-Candelas E Cragin C. Applied behavior analysis interventions for autism spectrum disorders. Brown Univ Child Adolesc Behav Lett. (2023) 39:1–5. 10.1002/cbl.30738

[2] McBain RK Kofner A Stein BD Cantor JH Vogt WB Yu H. State insurance mandates and the workforce for children with autism. Pediatrics. (2020) 146(4):e20193113. 10.1542/peds.2020-0836 PMC7546088 32900876

[3] Reichow B Hume K Barton EE Boyd BA. Early intensive behavioral intervention (EIBI) for young children with autism spectrum disorders (ASD). Cochrane Database Syst Rev. (2018) 5(5):CD009260. 10.1002/14651858.CD009260.pub3 29742275 PMC6494600

[4] Sandbank M Bottema-Beutel K Crowley LaPoint S Feldman JI Barrett DJ Caldwell N. Autism intervention meta-analysis of early childhood studies (project AIM): updated systematic review and secondary analysis. Br Med J. (2023) 383:e076733. 10.1136/bmj-2023-076733 37963634 PMC10644209

[5] Sandbank M Pustejovsky JE Bottema-Beutel K Caldwell N Feldman JI Crowley LaPoint S. Determining associations between intervention amount and outcomes for young autistic children: a meta-analysis. JAMA Pediatr. (2024) 178(8):763–73. 10.1001/jamapediatrics.2024.1832 38913359 PMC11197026

[6] Aguirre Mtanous NG Koenig J Nikahd M Effertz SE Silinonte S Hyer JM. Mental health outcomes associated with applied behavior analysis in a US national sample of privately insured autistic youth. Autism. (2026) 30(2):484–94. 10.1177/13623613251390604 41206741

[7] Willsey HR Willsey AJ Wang B State MW. Genomics, convergent neuroscience and progress in understanding autism spectrum disorder. Nat Rev Neurosci. (2022) 23(6):323–41. 10.1038/s41583-022-00576-7 35440779 PMC10693992

[8] Liu S Zeng D Yi C Zhu M. Neurocognitive mechanisms underlying autism spectrum disorders: a literature review. Front Psychiatry. (2025) 16:1633658. 10.3389/fpsyt.2025.1633658 41113188 PMC12532009

[9] Fountain C Winter AS Cheslack-Postava K Bearman PS. Developmental trajectories of autism. Pediatrics. (2023) 152(3):e2022058674. 10.1542/peds.2022-058674 37615073 PMC10551845

[10] Leadbitter K Buckle KL Ellis C Dekker M. Autistic self-advocacy and the neurodiversity movement: implications for autism early intervention research and practice. Front Psychol. (2021) 12:635690. 10.3389/fpsyg.2021.635690 33912110 PMC8075160

[11] Pellicano E Den Houting J. Shifting from “normal science” to neurodiversity in autism science. J Child Psychol Psychiatry. (2022) 63(4):381–96. 10.1111/jcpp.13534 34730840 PMC9298391

[12] Yang B Wang M Zhou W Wang X Chen S Potenza MN. Disrupted network integration and segregation involving the default mode network in autism spectrum disorder. J Affect Disord. (2023) 323:309–19. 10.1016/j.jad.2022.11.083 36455716

[13] Uddin LQ Supekar K Lynch CJ Khouzam A Phillips J Feinstein C. Salience network-based classification and prediction of symptom severity in children with autism. JAMA Psychiatry. (2013) 70(8):869–79. 10.1001/jamapsychiatry.2013.104 23803651 PMC3951904

[14] Williams ZJ Suzman E Bordman SL Markfeld JE Kaiser SM Dunham KA. Characterizing interoceptive differences in autism: a systematic review and meta-analysis of case-control studies. J Autism Dev Disord. (2023) 53(3):947–62. 10.1007/s10803-022-05656-2 35819587 PMC9832174

[15] Grove R Baillie A Allison C Baron-Cohen S Hoekstra RA. Empathizing, systemizing, and autistic traits: latent structure in individuals with autism, their parents, and general population controls. J Abnorm Psychol. (2013) 122(2):600–9. 10.1037/a0031919 23713510

[16] May KE Kana RK. Frontoparietal network in executive functioning in autism spectrum disorder. Autism Res. (2020) 13(10):1762–77. 10.1002/aur.2403 33016005

[17] Uddin LQ Supekar K Menon V. Reconceptualizing functional brain connectivity in autism from a developmental perspective. Front Hum Neurosci. (2013) 7:458. 10.3389/fnhum.2013.00458 23966925 PMC3735986

[18] Quadt L Garfinkel SN Mulcahy JS Larsson DEO Silva M Jones A-M. Interoceptive training to target anxiety in autistic adults (ADIE): a single-center, superiority randomized controlled trial. EClinicalMedicine. (2021) 39:101042. 10.1016/j.eclinm.2021.101042 34401684 PMC8350004

[19] Nuske HJ McGhee Hassrick E Bronstein B Hauptman L Aponte C Levato L. Broken bridges-new school transitions for students with autism spectrum disorder: a systematic review on difficulties and strategies for success. Autism. (2019) 23(2):306–25. 10.1177/1362361318754529 29458258

[20] Green SA Hernandez L Bookheimer SY Dapretto M. Salience network connectivity in autism is related to brain and behavioral markers of sensory overresponsivity. J Am Acad Child Adolesc Psychiatry. (2016) 55(7):618–26.e1. 10.1016/j.jaac.2016.04.013 27343889 PMC4924541

[21] Mandy W Murin M Baykaner O Staunton S Cobb R Hellriegel J. Easing the transition to secondary education for children with autism spectrum disorder: an evaluation of the systemic transition in education programme for autism Spectrum disorder (STEP-ASD). Autism. (2016) 20(5):580–90. 10.1177/1362361315598892 26304678 PMC4887819

[22] Maw KJ Beattie G Burns EJ. Cognitive strengths in neurodevelopmental disorders, conditions and differences: a critical review. Neuropsychologia. (2024) 197:108850. 10.1016/j.neuropsychologia.2024.108850 38467371

[23] Lai MC Anagnostou E Wiznitzer M Allison C Baron-Cohen S. Evidence-based support for autistic people across the lifespan: maximising potential, minimising barriers, and optimising the person-environment fit. Lancet Neurol. (2020) 19(5):434–51. 10.1016/S1474-4422(20)30034-X 32142628

[24] O'Hagan B Krauss SB Friedman AJ Bartolotti L Abubakare O Broder-Fingert S. Identifying components of autism friendly health care: an exploratory study using a modified Delphi method. J Dev Behav Pediatr. (2023) 44(1):e12–8. 10.1097/DBP.0000000000001139 36367772

[25] Hamilton LG Petty S. Compassionate pedagogy for neurodiversity in higher education: a conceptual analysis. Front Psychol. (2023) 14:1093290. 10.3389/fpsyg.2023.1093290 36874864 PMC9978378

[26] Nishith S O’Brien AM Li C Bungert L Oddis K Riddle J. Improving autistic experiences in the workplace: key factors and actionable steps. J Autism Dev Disord. (2025). 10.1007/s10803-025-07036-y 40991167

[27] Millar K Rodd C Rempel G Cohen E Sibley KM Garland A. The clinical definition of children with medical complexity: a modified Delphi study. Pediatrics. (2024) 153(6):e2023064556. 10.1542/peds.2023-064556 38804054

[28] Lin E Noritz G Agrawal R Bell DS Foster JEA. Home health care of children, adolescents and young adults with complex medical needs: clinical report. Pediatrics. (2025) 156(3):e2025073171. 10.1542/peds.2025-073171 40850691

[29] Solnit R. What technology takes from us, and how to take it back. The Guardian [Internet] (2026). Available online at: https://www.theguardian.com/news/ng-interactive/2026/jan/29/what-technology-takes-from-us-and-how-to-take-it-back (Accessed March 6, 2026).

[30] Merahn S. Knowledge representation and care planning for population health management. J Med Pract Manage. (2015) 31(2):126–30. 26665485

[31] Gordon RS Jr. An operational classification of disease prevention. Public Health Rep. (1983) 98(2):107–9. 6856733 PMC1424415

[32] Delgadillo J Ali S Fleck K Agnew C Southgate A Parkhouse L. Stratified care vs stepped care for depression: a cluster randomized clinical trial. JAMA Psychiatry. (2022) 79(2):101–8. 10.1001/jamapsychiatry.2021.3539 34878526 PMC8655665

[33] Bodenheimer T Wagner EH Grumbach K. Improving primary care for patients with chronic illness. J Am Med Assoc. (2002) 288(14):1775–9. 10.1001/jama.288.14.1775 12365965

[34] Merahn S. Treating invisible wounds: the case for trauma-informed care in autism. J Am Acad Child Adolesc Psychiatry. (2026) S0890-8567(26)00024-9. 10.1016/j.jaac.2026.01.016 41638425

